# Folate and Nutrients Involved in the 1-Carbon Cycle in the Pretreatment of Patients for Colorectal Cancer

**DOI:** 10.3390/nu7064318

**Published:** 2015-06-02

**Authors:** Ariana Ferrari, Aline Martins de Carvalho, Josiane Steluti, Juliana Teixeira, Dirce Maria Lobo Marchioni, Samuel Aguiar

**Affiliations:** 1 Department of Pelvic Surgery, A.C. Camargo Cancer Center, Rua Professor Antônio Prudente, 211, Liberdade, São Paulo (SP) CEP 01509-010, Brazil; E-Mail: samuel.aguiar.jr@gmail.com; 2 Department of Nutrition, School of Public Health, University of São Paulo, Av. Dr. Arnaldo 715, Consolação, São Paulo (SP) CEP 01246-904, Brazil; E-Mails: alinemartins2011@gmail.com (A.M.); jsteluti@gmail.com (J.S.); jwliana_at@yahoo.com.br (J.T.); dirce.marchioni@gmail.com (D.M.L.M.)

**Keywords:** folate, 1-carbon cycle, colorectal carcinogenesis.

## Abstract

To assess the ingestion of folate and nutrients involved in the 1-carbon cycle in non-treated patients with colorectal adenocarcinoma in a reference center for oncology in southeastern Brazil. In total, 195 new cases with colorectal adenocarcinoma completed a clinical evaluation questionnaire and a Food Frequency Questionnaire (FFQ). Blood samples from 161 patients were drawn for the assessment of serum folate. A moderate correlation was found between serum concentrations of folate, folate intake and the dietary folate equivalent (DFE) of synthetic supplements. Mulatto or black male patients with a primary educational level had a higher intake of dietary folate. Of patients obtaining folate from the diet alone or from dietary supplements, 11.00% and 0.10%, respectively, had intake below the recommended level. Of the patients using dietary supplements, 35% to 50% showed high levels of folic acid intake. There was a prevalence of inadequacy for vitamins B2, B6 and B12, ranging from 12.10% to 20.18%, while 13.76% to 22.55% of patients were likely to have adequate choline intake. The considerable percentage of patients with folate intake above the recommended levels deserves attention because of the harmful effects that this nutrient may have in the presence of established neoplastic lesions.

## 1. Introduction

The incidence of colorectal cancer (CRC) is increasing in Brazil, especially in the major metropolitan regions of the Southeast, probably due to modifications in lifestyle habits [1]. The populations in emerging countries have complex patterns of nutritional status, with some areas acquiring the lifestyle and nutrition patterns of developed countries and others maintaining the nutritional characteristics associated with developing countries.

Epidemiological studies have shown the importance of folate in colorectal carcinogenesis due to its key role in the methylation and synthesis of nucleotides [2]. B-complex vitamins, such as vitamins B2, B6 and B12 [3], choline, and betaine [4] act as cofactors in the reactions of 1-carbon metabolism and are therefore essential for the metabolic processes involving folate.

Because folate intake has a controversial but important association with colorectal carcinogenesis, we investigated the pattern of folate intake and other associated nutrients in a cohort of CRC patients from a single institution in Southeast Brazil.

The objectives of this study were to assess the ingestion of folate and other nutrients involved in the 1-carbon cycle in untreated patients with colorectal adenocarcinoma at an oncology referral center in southeastern Brazil and to identify clinical variables that are associated with folate serum levels and intake.

## 2. Methodology

### 2.1. Study Design

This was an observational, cross-sectional study with prospective data collection of new cases of patients diagnosed with adenocarcinoma of the colon and rectum at the Colorectal Tumor Center of A.C. Camargo Cancer Center from May 2011 to May 2012. The inclusion criteria included patients with adenocarcinoma of the colon or rectum, at any stage of the disease and with an indication for surgical intervention at the primary site, or patients with adenomatous lesions and an indication for surgical intervention. Patients who had previously undergone surgery, radiotherapy and/or chemotherapy for colorectal tumor; patients with colon or rectal tumor recurrence; patients previously treated with chemotherapy for another malignancy in the last 3 months; or patients who, during the interview, did not present clinical conditions and/or an understanding when completing the questionnaires were excluded.

### 2.2. Clinical Evaluation Questionnaire

The questionnaire consisted of sociodemographic (sex, age, race, and educational level) and clinical (tumor site, clinicopathological staging) questions. In total, 195 patients were evaluated. The system used for clinicopathological staging was based on the Cancer Staging Manual published by the American Joint Committee on Cancer (AJCC) [5].

### 2.3. Dietary Assessment

A dietary assessment was conducted for alcohol, folate, vitamin B2, vitamin B6, vitamin B12, choline, betaine, methionine, energy, carbohydrate, protein and lipid using a validated Food Frequency Questionnaire (FFQ) by LAMEZA (2010) [6]. Of the 195 patients who participated in the study, 189 were included based on their dietary intake, with 169 patients obtaining folate from their diet alone and 20 patients using supplements containing folic acid. The values obtained from the FFQ were converted to energy and nutrient intake values through the software *Nutrition Data System for Research-NDSR* [7] and were considered unadjusted habitual food intake values (the raw data). Dietary synthetic folate values were corrected according to a mandatory fortification of wheat flour and corn (150 μg of folic acid (100 g)^−1^ of flour) that has been implemented in Brazil since 2004 [8]. In addition, corrections were made for the differences in the additive amount of folic acid in fortified foods (150 μg (100 g)^−1^ of flour in Brazil and 140 μg (100 g)^−1^ of flour in the United States). After these corrections, and taking into account the differences in the bioavailability of folate found naturally in food and folic acid in fortified foods (1 μg synthetic folate = 1.70 μg folate) [9], DFE was calculated. In addition to the DFE values from diet, the values of folic acid intake through supplementation (synthetic folate supplement) were evaluated. In this case, the patients were asked whether they consumed multivitamins. If yes, the value of folic acid was calculated for each supplement. Only the amount of folic acid in the supplements was considered. From this value, we calculated the DFE for the supplements, assuming that every 1 μg of folic acid supplement on an empty stomach provides 2.0 μg of DFE [10]. The overall DFE was calculated from the sum of DFE from the diet and DFE from supplements. In total, 20 patients used supplements with folic acid. After calculating the unadjusted values, the data were calibrated. For calibration, we used three 24-h dietary recalls and a second FFQ collected in a previous study [6]. The R24 data were used as a reference and subjected to linear regression, with β1 values used as a calibration factor for the FFQ data collected. To assess the prevalence of folate inadequacy, patients were divided into two groups. The first group was composed of patients who ingested this nutrient from diet alone (*n* = 169), and the second group consisted of patients who ingested folate through diet and supplementation (*n* = 20). For patients who ingested folate from diet alone, an Estimated Average Requirement (EAR) cutoff value of 20 μg day^−1^ and a Tolerable Upper Intake Levels (UL) of 1000 μg day^−1^ were used, according to equations proposed by FISBERG *et al.* (2005) [11]. Patients on a diet with folic acid supplementation (*n* = 20) were treated individually. In this case, we assessed whether there were any patients with food intake below the RDA cutoff point of 400 μg day^−1^. For intake above the UL, a qualitative interpretation was performed and the percentage of patients above or below the UL was calculated [12]. For all patients, the prevalence of inadequate intake and the unadjusted and calibrated values for vitamin B2, vitamin B6 and vitamin B12 were also based on the EAR cutoff value [10]. There are no EAR recommendations for choline, therefore all values were considered [13].

### 2.4. Determination of Serum Folate

After 4 h of fasting, 10-mL blood samples were drawn from the patients by preoperative venipuncture. For patient candidates who were eligible for neoadjuvant treatment, samples were drawn from peripheral blood before radiochemotherapy. As previously described by Pufulete *et al.* [14], the competitive enzyme immunoassay technique was used for the analysis of the serum folic acid concentration. Of the 195 patients who participated in the study, 161 were examined for serum folic acid.

### 2.5. Ethical Aspects

This study was approved by the research ethics committee of Fundação Antonio Prudente under number 1542/11.

## 3. Results

The median age was 61 years (Quartile interval _25%–75%_: 53–71 years). Of the 195 patients interviewed, 94 (48.21%) were female and 101 (51.79%) were male. Of the patients evaluated, 63 (32.31%) completed their primary education, 59 (30.26%) attained a high school/college incomplete level of education and 73 (37.44%) were at a college graduate/postgraduate level of education. There was a predominance of white patients (70.26%), followed by Asian (17.95%) and mulatto/black (11.79%). There was a higher incidence of colon tumors (66.67%) compared with rectal tumors (33.33%). In clinicopathological staging, 50.77% of the patients conformed to staging I and II and 47.69% to staging III and IV.

Of the 195 patients interviewed, 189 were included in the dietary analysis. Table 1 presents the mean, standard deviation and minimum and maximum nutrient values obtained from the unadjusted and calibrated FFQ.

**Table 1:** Mean, standard deviation and minimum and maximum nutrient values from the unadjusted and calibrated FFQ in the pretreatment of patients for colorectal adenocarcinoma.

Nutrients FFQ (*n* = 189)		Mean	SD	Minimum	Maximum
Energy (Kcal)	unadjusted	3144.58	1119.22	1120.72	6312.25
	calibrated	1766.32	171.13	1351.40	2160.68
Lipid (g)	unadjusted	103.35	40.29	38.48	242.95
	calibrated	68.46	8.92	49.60	93.22
Carbohydrate (g)	unadjusted	434.48	208.84	69.28	1280.54
	calibrated	204.79	20.54	140.09	263.48
Protein (g)	unadjusted	122.31	50.06	50.29	304.53
	calibrated	79.33	6.60	66.56	97.49
Alcohol (g)	unadjusted	8.41	21.90	0.00	214.99
	calibrated	0.41	0.41	0.00	2.42
Vitamin B2 (mg)	unadjusted	2.46	0.89	0.78	5.46
	calibrated	1.55	0.09	1.29	1.79
Vitamin B6 (mg)	unadjusted	2.95	1.70	0.78	12.28
	calibrated	1.51	0.22	1.02	2.41
Vitamin B12 (μg)	unadjusted	7.75	3.90	1.38	22.14
	calibrated	4.40	0.81	2.37	6.71
Methionine (g)	unadjusted	2.71	1.22	1.02	7.22
	calibrated	1.81	0.19	1.43	2.37
Natural folate (μg)	unadjusted	376.18	167.80	95.59	1075.83
	calibrated	215.65	24.60	151.51	291.54
Synthetic folate (μg)	unadjusted	146.61	90.52	10.77	549.99
	calibrated	76.86	12.70	40.23	112.61
DFE diet (μg)	unadjusted	625.43	257.29	116.26	1584.56
	calibrated	361.25	13.58	311.79	396.00
Choline (mg)	unadjusted	449.85	184.52	178.73	1140.15
	calibrated	284.54	25.22	233.95	356.02
Betaine (mg)	unadjusted	255.58	198.69	23.98	1692.13
	calibrated	130.63	9.50	100.38	167.31

DFE—Dietary Folate Equivalent.

One-hundred and sixty-one patients were examined for serum folate levels. The mean serum folate was 10.89 ng mL^−1^. When the median serum folate concentrations were compared according to sociodemographic variables, there was a significant difference (*p* = 0.001) between females (12.95 ng mL^−1^) and males (10.10 ng mL^−1^). In addition, patients aged less than 61 years had a median serum folate concentration that was significantly lower than the group of patients ≥61 years (10.45 *vs.* 11.60 ng mL^−1^), with a *p*-value = 0.04. The concentrations of serum folate showed no significant differences in relation to race, education level, tumor site and staging.

To evaluate the correlation between the values of folate intake and the level of serum folate, Spearman’s rank correlation coefficient test was used, as shown in Table 2. Of the 189 patients who were included in the dietary analysis, 156 were examined for serum folate levels. Of the 20 patients who consumed folic acid supplements, 17 were examined for serum folate levels. We observed a moderate correlation between the intake of synthetic folate supplement and serum folate levels. The same moderate correlation was observed between DFE supplement values and serum folate; both showed a significant difference (*p* = 0.02). There was a fair correlation between the total calibrated DFE values and serum folate levels, but this was not significant (*p* = 0.06). There was no correlation between the serum folate and intake values for vitamin B2, B6, and B12; methionine; choline; betaine; and alcohol.

Table 3 shows a comparison of the median DFE from unadjusted and calibrated diet data according to the sociodemographic and clinical characteristics. Women had a significantly lower intake of DFE compared with men, with a median unadjusted diet DFE for women and men of 537.09 μg and 610.29 μg, respectively (*p* = 0.03). The same *p*-value was found in the comparison of median calibrated diet DFE among women (358.67 μg) and men (362.89 μg). The unadjusted and calibrated DFE values were highest among the mulatto/black race, followed by the white and Asian races. The difference was significant between the Asian and white races and between the Asian and mulatto/black races (*p* = 0.03). Regarding the educational level, patients completing their primary education had an unadjusted and calibrated DFE intake that was significantly higher than those at the college graduate/postgraduate level (*p* = 0.01). The unadjusted and calibrated DFE values showed no significant difference in relation to age.

**Table 2:** Spearman’s rank correlation coefficient of folate intake with the dosage of serum folate in the pretreatment of patients for colorectal adenocarcinoma.

	Serum Folate (ng mL^−1^)		
	*N*	Spearman correlation coefficient (rho)	*p*
Unadjusted DFE diet ^1^ (μg)	156	0.05	0.53
Calibrated DFE diet ^1^ (μg)	156	0.05	0.53
Synthetic folate supplement	17	0.54	0.02 *
DFE supplement ^2^ (μg)	17	0.54	0.02 *
Total unadjusted DFE ^3^ (μg)	17	0.40	0.10
Total calibrated DFE ^3^ (μg)	17	0.45	0.06

DFE—Dietary Folate Equivalent; ^1^ DFE diet = natural folate + 1.7 × (dietetic synthetic folate); ^2^ DFE supplement = synthetic folate supplement × 2; ^3^ Total DFE = DFE diet + DFE supplement; * *p* < 0.05.

**Table 3:** Median comparison of DFE from unadjusted diet data and calibrated diet DFE according to sociodemographic and clinical characteristics in the pretreatment of patients for colorectal adenocarcinoma.

	DFE Diet ^1^ Unadjusted (μg) (*n* = 189)			DFE Diet ^1^ Calibrated (μg) (*n* = 189)		
	*N*	Median	*p*	*N*	Median	*p*
**Sex**						
Female	93	537.09	0.03 *	93	358.67	0.03 *
Male	96	610.29		96	362.89	
**Age**						
<61 years	86	615.20	0.17	86	363.15	0.17
≥61 years	103	560.96		103	360.10	
**Race**						
Asian (a)	35	495.93	0.03 *^ab,ac^	35	356.06	0.03 *^ab,ac^
White (b)	135	591.95		135	361.88	
Mulatto/Black (c)	19	620.98		19	363.47	
**Education level**						
Primary education (a)	62	633.04	0.01 *^ac^	62	364.10	0.01 *^ac^
High school/college incomplete level (b)	55	598.83		55	362.26	
College graduate/postgraduate level (c)	72	524.09		72	357.86	
**Tumor site**						
Colon	125	584.12	0.42	125	361.44	0.42
Rectal	64	610.29		64	362.89	
**Staging**						
I e II	97	584.70	0.41	97	361.47	0.41
III e IV	89	589.11		89	361.72	

DFE—Dietary Folate Equivalent; ^1^ DFE diet = natural folate + 1.7 × (dietetic synthetic folate); * *p* < 0.05; In race, “a” is Asian, “b” is white and “c” is mulatto/black; In Education level, “a” is primary education, “b” is high school and “c” is college graduate.

Regarding the intake of folic acid supplements, a comparison of the medians of supplement, total unadjusted and total calibrated DFE is shown in Table 4, according to the sociodemographic characteristics. DFE supplement intake was significantly higher among women (1750.00 μg) compared to men (440.00 μg). DFE supplement values showed no significant difference in relation to age, race and education level. Similar results were obtained for the total unadjusted and calibrated DFE, where the median values showed no significant difference in relation to sociodemographic variables. As previously mentioned, of the 189 patients evaluated, 10.58% received folic acid supplementation, and 8.46% of these had colon cancer and 2.12% had rectal cancer. There was an association between total unadjusted and calibrated DFE with the tumor site. The median total unadjusted DFE intake of patients with colon cancer was 1177.18 μg *vs.* 845.60 μg for rectal tumor patients (*p* = 0.001). The total calibrated DFE intake was also higher among colon tumor patients when compared with individuals with rectal tumors, with a median intake of 845.81 μg in patients with colon tumors and 702.72 μg in patients with rectal cancer (*p* = 0.02) (Table 5).

Regarding the prevalence of folate inadequacy in patients with intake from diet alone, 11.00% had intakes below the EAR according to the unadjusted FFQ. However, according to the calibrated FFQ, only 0.10% of individuals had intakes below the recommended EAR level. When evaluating the prevalence of inadequacy above the UL, 6.06% of patients had a dietary folate intake above the UL according to the unadjusted FFQ, while the calibrated FFQ showed no cases of inadequacy.

Of the patients on diets with folic acid supplementation, no patient had an intake below the cutoff points of the Recommended Dietary Allowances (RDA). However, when considering the values of the UL through a qualitative interpretation of the adequacy of folate intake, the FFQ evaluates the usual intake and thus corresponds to a survey over a large number of days. According to unadjusted and calibrated FFQ, 50.00% and 35.00% of patients, respectively, had a greater intake up to the UL, which is a potential risk for adverse effects.

The prevalence of inadequacy of vitamins B2, B6 and B12 was calculated using the values of EAR and is presented in Table 6. We observed a higher prevalence of inadequate intake of vitamin B6, followed by vitamin B12, and B2. For the evaluation of inadequate choline, a qualitative evaluation was used, which considered that the FFQ takes into account a greater number of days. When evaluated as the unadjusted FFQ, 22.55% of female patients and 13.76% of males had intake values of choline above the levels stated by the AI. This indicates that the mean intake in these individuals is likely adequate. Nevertheless, when analyzed using the calibrated FFQ, no patient had a dietary intake above the AI and the adequacy of intake could not be determined.

## 4. Discussion and Conclusion

This study is one of the few published studies that present the dietary intake of folate and nutrients involved in the 1-carbon cycle in CRC patients without any previous treatment.

A study conducted with 196 patients with CRC, which used a FFQ validated for Portuguese individuals, found a mean intake of vitamin B6, vitamin B12, folate, methionine and alcohol of 2.80 mg day^−1^ (SD = 1.06), 14.50 μg day^−1^ (SD = 9.10), 401.60 μg day^−1^ (SD = 161.9) 2.85 mg day^−1^ (SD = 1.28) and 25.17 g day^−1^ (SD = 39.80), respectively [15]. These results are consistent with the present study with regard to the unadjusted data for B6 and methionine and the calibrated data for folate. Jiang *et al.* [16] assessed the relationship between folate, methionine and alcohol and a genetic polymorphism and found a mean folate intake similar to the unadjusted data found in this study, 634 μg day^−1^ (SD = 307) for patients with colon cancer and 638 μg day^−1^ (SD = 334) for rectal tumors. In the same study, patients with colon cancer had a mean energy intake of 4260 Kcal day^−1^ (SD = 2220) and methionine of 2047 mg day^−1^ (SD = 939). In patients with rectal cancer, the mean energy intake was 4321 Kcal day^−1^ (SD = 1669) and methionine was 2117 mg day^−1^ (SD = 801). In another study conducted with 787 patients with colorectal tumors, the mean energy intake was 1588.6 Kcal (SD = 449.5) and folate was 248.1 μg day^−1^ (SD = 111.1). Furthermore, 54.5% of these patients consumed <5 g day^−1^ of alcohol, 19.10% consumed from 5 to <30 g day^−1^ and 26.40% consumed alcohol in quantities greater than 30 g day^−1^ [17]. Laso *et al.* [18] evaluated the dietary intake of 246 patients with CRC and found a mean intake of vitamin B2 of 1.91 mg day^−1^ (SD = 0.6), vitamin B6 of 1.98 mg day^−1^ (SD = 0.5), vitamin B12 of 7.67 mg day^−1^ (SD = 4.7), folate of 288.1 μg day^−1^ (SD = 89.1) and alcohol of 9.94 g day^−1^ (SD = 18.4). These data are similar to the unadjusted data found in this study in relation to vitamin B12 and alcohol. Al-ghnaniem *et al.* [19] described in their study a mean alcohol intake of 13 g day^−1^ and a mean folate intake of 289 μg day^−1^. The mean folate intake in the study of Pufulete *et al.* [14] was 304 μg day^−1^.

An important difference between this study and previous studies is that folate intake in this study was assessed by several methods, including an assessment of the values from the diet (DFE diet), from supplementation by fortification and supplements (DFE supplement), and from dietary folate plus supplemental folate (DFE total). We found only one study in the literature that considered these three types of folate. This study suggests that the best way to assess folate intake is through the total DFE because it encompasses the 19 different sources of folate [20].

FFQ are one of the most commonly used methods for evaluating habitual dietary intake in large-scale epidemiological studies, given their low cost and ease of application [21,22]. However, the errors present in the measurements of the questionnaire may attenuate the estimates of the relative risks that are found, and thus diminish the statistical power of studies evaluating the relationship between diet and disease [22,23]. Knowing that error is inherent in food intake measurements, methodological strategies have been used in an attempt to make the measurements obtained through the FFQ closer to the quantities actually consumed, which are the calibration. To assess the folate intake FFQ was used and fasting serum folate levels. FFQ relies on reported food intake and although it is a validated tool, it is prone to error, using the 24h as reference method only partly removes this error; indeed it is easier to accurately estimate folate intake in subjects who use supplements (as evidenced by the results presented in Table 2).

Biochemical markers are more sensitive and specific when compared to methods of dietary intake assessment through questionnaires and/or dietary recalls [11]. The study of Al-ghnaniem *et al.* [19] showed a mean serum folate of 12.30 ng mL^−1^, a mean level greater than that found in the present study. In contrast, some studies have shown lower plasma levels compared to this study. For example, Pufulete *et al.* [14] found a mean serum folate value of 5.40 ng mL^−1^ and Chang *et al.* [24] found a level of 5.02 ng mL^−1^ (SD = 4.43 ng mL^−1^).

**Table 4:** Comparison of the medians of supplement, total unadjusted and total calibrated DFE according to sociodemographic characteristics in the pretreatment of patients for colorectal adenocarcinoma.

	DFE Supplement ^1^ (μg) (*n* = 20)			Total Unadjusted DFE ^2^ (μg) (*n* = 20)			Total Calibrated DFE ^2^ (μg) (*n* = 20)		
	*N*	Median	*p*	*N*	Median	*p*	*N*	Median	*p*
**Sex**									
Female	12	1750.00	0.01 *	12	2724.50	0.44	12	2128.77	0.05
Male	8	440.00		8	969.77		8	794.31	
**Age**									
<61 years	3	304.00	0.55	3	900.26	0.63	3	659.87	0.49
≥61 years	17	480.00		17	1069.11		17	837.44	
**Race**									
Asian	6	480.00	0.22	6	984.68	0.16	6	835.98	0.22
White	13	480.00		13	997.18		13	831.99	
Mulatto/Black	1	20,000.00		1	21,079.60		1	20,382.30	
**Education level**									
Primary education	8	8000.00	0.07	8	9150.01	0.06	8	8382.11	0.09
High school/college incomplete level	4	480.00		4	956.48		4	834.66	
College graduate/postgraduate level	8	440.00		8	910.92		8	793.44	

DFE—Dietary Folate Equivalent; ^1^ DFE supplement = synthetic folate supplement × 2; ^2^ Total DFE = DFE diet + DFE supplement.

**Table 5:** Comparison of the medians of supplement, total unadjusted and total calibrated DFE according to clinical characteristics in the pretreatment of patients for colorectal adenocarcinoma.

	DFE Supplement ^1^ (μg) (*n* = 20)			Total Unadjusted DFE ^2^ (μg) (*n* = 20)			Total Calibrated DFE ^2^ (μg) (*n* = 20)		
	*N*	Median	*p*	*N*	Median	*p*	*N*	Median	*p*
**Tumor site**									
Colon	16	480.00	0.06	16	1177.18	0.001 *	16	845.81	0.02 *
Rectal	4	352.00		4	845.60		4	702.72	
**Staging**									
I e II	12	480.00	0.96	12	1059.72	0.58	12	834.71	0.93
III e IV	8	440.00		8	1005.74		8	799.17	

DFE—Dietary Folate Equivalent; ^1^ DFE supplement = synthetic folate supplement × 2; ^2^ Total DFE = DFE diet + DFE supplement; * *p* < 0.05.

**Table 6:** The prevalence of inadequacy of vitamins B2, B6, B12 and folate in the pretreatment of patients for colorectal adenocarcinoma.

	% Bellow of EAR ( *n* = 189)
Unadjusted FFQ	
Vitamin B2	4.00
Vitamin B6	14.86
Vitamin B12	7.00
Folate	11.00
Calibrated FFQ	
Vitamin B2	0.00
Vitamin B6	20.18
Vitamin B12	0.10
Folate	0.10

FFQ—Food Frequency Questionnaire; EAR—Estimated Average Requirement.

In the present study, there was a significant difference in plasma levels of folate in relation to sex and age, but no studies have been found in the literature that evaluated this relationship. Furthermore, when assessing the correlation of serum folate concentrations with folate intake, a significant difference was found in relation to a synthetic folate from supplement as well as a DFE supplement. Additionally, there were no verified studies that assessed the correlation between the ingestion of supplements with plasma folate levels.

The present study also evaluated the correlation between serum folate with other nutrients involved in the 1-carbon cycle, but significant differences were not found. Studies were not found in the literature that assessed this correlation. However, in relation to dietary data, one study showed a moderate correlation between vitamin B2 and B6 (rho = 0.44) and folate (rho = 0.45). In that study, vitamin B6 showed a moderate correlation with vitamin B12 (rho = 0.38) and folate (rho = 0.48). Vitamin B12 also showed a moderate correlation with folate (rho = 0.34) [25].

In this study, men ingested significantly more folate (unadjusted and calibrated) than women. In addition, Asian participants ingested less folate, both unadjusted and calibrated, than the participants of any other race. The difference was significant between the Asian and white races and between the Asian and mulatto/black races. Regarding education, patients with up to a primary school education had an unadjusted and calibrated folate intake that was significantly higher than patients with higher education at the college graduate/postgraduate level. We found no studies in the literature that showed a correlation between folate intake with the variables mentioned here.

The prevalence of the inadequacy of a particular nutrient has been a target of interest among researchers because this information allows for the development of healthcare strategies, such as planning and monitoring actions that are specific to a group of individuals [26]. Folate, vitamins B2, B6, B12, and choline are part of numerous biochemical reactions that are involved in the 1-carbon cycle, which is essential for the synthesis and methylation of DNA, the synthesis of RNA precursors and the conversion of homocysteine in methionine [14,27,28,29]. A deficiency of nutrients in this group can lead to decreased levels S-adenosylmethionine (SAM), chromosomal instability, changes in transcriptional regulation, poor incorporation of uracil into DNA, *DNA* hypomethylation and increased risk of colorectal tumors [30,31,32,33,34,35]. When analyzing the prevalence of folate inadequacy in patients with intake from diet alone, 11.00% and 0.10% had intakes below the EAR according to the unadjusted and calibrated FFQ, respectively. One of the mechanisms by which folate deficiency can lead to CRC is related to the synthesis of purines and thymidylate. Thus, adequate levels are essential for the synthesis, stability, integrity and adequate repair of DNA [29,36]. In some studies, a dietary intake of 400 μg day^−1^ of folate for 10 weeks increased DNA methylation in lymphocytes and in the colonic mucosa of patients with colorectal adenomas [37,38]. One study that evaluated the colon in 609 cases showed that an intake of <200 μg day^−1^ of folate was related to increased hypomethylation of LINE-1 regulatory sequences in contrast to a dietary intake of folate ≥400 μg day^−1^, which showed a decrease in hypomethylation. The consumption of ethanol ≥15 g day^−1^ was associated with an increased risk of LINE-1 hypomethylation [39].

Regarding vitamin B6, the prevalence of inadequacy according to unadjusted and calibrated FFQ was 14.86% and 20.18%, respectively. Vitamin B6 acts as an enzyme cofactor for serine hydroxy-methyltransferase (SHMT) and cystathionine b-synthase (CBS). Vitamin B6 is responsible for the formation of glycine and 5,10-methylenetetrahydrofolate and allows for the irreversible reaction of homocysteine to cystathionine [40]. It is known that vitamin B6 deficiency can lead to hyperhomocysteinemia, weakness, nervous disorders, irritability, insomnia and difficulty walking [41].

The prevalence of inadequate dietary intake of vitamin B12 was 7.00% and 0.10% according to unadjusted and calibrated FFQ, respectively. During the methylation of homocysteine to methionine, vitamin B12 acts as a cofactor for the enzyme methionine synthase [42,43]. Additionally, this vitamin plays an important role during the isomerization of l-methylmalonyl-CoA to succinyl-CoA [44].

Regarding vitamin B2, the prevalence of inadequacy was 4%, assessed by the FFQ unadjusted data only. Vitamin B2, besides taking part in the remethylation of homocysteine to methionine, acts as a cofactor for methylenetetrahydrofolate reductase (MTHFR) and pyridoxine 5′-phosphate oxidase [45].

The prevalence of the inadequacy of choline could only be assessed by the unadjusted FFQ and was 22.55% in female patients. Of these female patients, 13.76% likely had adequate intake. This nutrient participates as a cofactor for SAM-dependent transmethylation reactions [46].

A low proportion of patients showed folate deficiency in this sample. On the other hand, a high proportion of patients showed folate intake above the recommended levels. Female patients and patients less than 61 years old presented significantly lower serum folate levels.

In the present study, we found a considerable number of patients who had a folate intake above the UL, both those who only ingested this nutrient from the diet and, in many cases, those patients with supplementation. Food fortification with folic acid has been deployed in several countries around the world to prevent embryonic neural tube defects and some diseases, such as cancer [47], and is likely the most important action in the field of nutrition and public health [48]. After fortification in the U.S. and Canada, considerable increases have been seen both in the intake and serum concentration of folic acid [49,50]. However, folate fortification or folic acid supplementation may negatively interfere with the 1-carbon cycle and thus could become an important issue for extrapolation [51]. Folate intake above the UL, in addition to not diminishing the risk for CRC, increases the risk that an individual may develop this type of tumor [52]. Studies using animal models have shown that before the presence of neoplastic foci, a moderate deficiency of dietary folate increased the onset and progression of adenomas, whereas supplementation with 4 to 10 times above the basal daily requirement for folic acid suppressed the onset and progression of these adenomas. However, when such folate intervention was performed after the establishment of preneoplastic lesions, a moderate deficiency of dietary folate suppressed tumor growth and progression in addition to promoting the regression of the tumor [53,54]. A study conducted by Lindzon *et al.* which aimed to evaluate the effects of folic acid supplementation in aberrant crypt foci and CCR, used 152 male rats at weaning that received supplementation of 2 mg folic acid (kg day)^−1^. Six weeks after the induction of the aberrant crypt foci, the rats were randomized into four groups. In these groups, the rodents received 0, 2, 5 or 8 mg (kg day)^−1^ of folic acid. The rats were sacrificed after 34 weeks to assess the result of supplementation. The number of aberrant crypt foci increased with increasing amounts of supplementation. Furthermore, although the tumor incidence was significantly different among the four groups from the tumor multiplicity, the tumor load and rectal epithelial proliferation were positively correlated with the folate levels and inversely correlated with the concentration of homocysteine [55]. Thus, studies in animals have evaluated the effects of supplements [56,57]. For this reason, recent studies in humans suggest that in normal mucosa, folic acid can prevent the onset of CRC. Despite not being completely understood because of its dual role, folate intake in a previously established preneoplastic lesion can accelerate the growth of tumor cells [36,58,59,60,61]. Thus, it is stressed that careful assessment should be given with regards to the dose and when to start folate intervention, while observing the presence or absence of preneoplastic lesions [36,59]. This is because folate appears to have a dual modulating role in colorectal cancer (CCR), which involves the onset and progression of this tumor and depends on the dose and start of intervention [36]. The mechanisms by which this occurs include the provision of precursors for DNA synthesis and hypermethylation of tumor suppressor genes [29].

In the study by Kim *et al.* [17], 18.00% of the patients used multivitamin supplements. Two other studies that evaluated 28 and 18 patients with colorectal tumors found that 93% [19] and 7% [20] of these patients ingested supplementation, respectively. Corroborating the present work, one study reported a higher intake of multivitamins for patients with colon cancer, 6.20% of patients with rectal cancer and 9.10% of patients with colon cancer [62].

Recent studies have shown that folate deficiency in normal intestinal mucosa can lead to the instability and incorporation of uracil in the DNA molecule. Therefore, adequate nutritional intake of folate can act as a protective agent against cancer in the carcinogenesis of the colon and rectum. However, in preneoplastic lesions, with intense cell division, folate deficiency appears to disrupt this process, thus inhibiting tumor growth and even tumor regression [57]. This occurs because folic acid can act as a substrate for tumor growth and replication, increasing the chances of disease progression [29,63,64].

Several authors have shown no or positive associations between folate supplementation and the recurrence of adenomas [25,63,65,66]. In study by Cole *et al.* [65], the participants were randomly chosen and 516 received 1 mg day^−1^ of folic acid and 505 received placebo. They also were separately randomized to receive aspirin (81 or 325 mg day^−1^) or placebo. Follow-up consisted of two colonoscopic surveillance cycles, the first after three years and the second between three and five years, and found that patients who received folic acid at 1 mg day^−1^ did not have reduced colorectal adenoma risk. Logan *et al.* [66], in a randomized study, noted that aspirin (300 mg day^−1^) but not folate (0.5 mg day^−1^) use was found to reduce the risk of colorectal adenoma recurrence. Sauer *et al.* [51], notes that fortification or supplementation with folic acid can interfere negatively in the carbon-1 cycle and thus becomes an important issue for extrapolation. According to Bollheimer *et al.* [52], folate intake above the UL, in addition to not reducing the risk for CRC, increases the risk that the individual will develop this type of tumor [52].

After mandatory fortification in Brazil, dietary folate intake probably increased in the population. Steluti *et al.* (2011) conducted a study in Brazil in order to investigate serum concentrations and the prevalence of inadequate folate intake and also vitamin B6 and vitamin B12 intakes. The study showed low prevalence rates of inadequate folate; vitamin B6 and vitamin B12 intakes were low, which is possibly the result of improved access to and availability of foods that are dietary sources of these vitamins [67]. In addition, a recent study in Brazil, analyzed folic acid intake before and since mandatory fortification and showed that prevalence of inadequate folic acid intake mainly decreased in adolescents and adult males. The paper also discusses that while folate has been associated with decreased risk of certain chronic diseases, there is strong evidence that the excess of nutrients may increase DNA synthesis, stimulating cell proliferation and participate in tumor progression [68]. Furthermore, because current evidence of the benefits of regular use and risk of excessive consumption of supplements containing folic acid, it is necessary to monitor use of supplements [69,70].

## 5. Conclusion

The purchase of multivitamins by the population is often performed without guidance. Moreover, due to the difficulty of the early diagnosis of CRC, the medical professional and/or nutritionist cannot adequately guide the patient in the use of nutritional supplements. Thus, because of the increased supply of folic acid through foods, in combination with the use of multivitamin supplements, a part of the population may far exceed the intake of folic acid recommended by the DRIs, which established a tolerable UL of 1 mg day^−1^. More studies are needed to understand the impact of high folic acid intake through food fortification and use of dietary supplements, as well as other nutrients involved in the 1-carbon cycle, and whether high intake promotes adverse health effects, especially in cancer patients. We also suggest further studies to identify potential polymorphisms in the MTHFR enzyme, which is involved in the metabolic pathways that degrade homocysteine, in order to determine whether changes in this enzyme can effectively interfere with folate metabolism.
